# Terminal cationization of poly(*N*-isopropylacrylamide) brush surfaces facilitates efficient thermoresponsive control of cell adhesion and detachment

**DOI:** 10.1080/14686996.2021.1929464

**Published:** 2021-06-22

**Authors:** Masamichi Nakayama, Tomonori Kanno, Hironobu Takahashi, Akihiko Kikuchi, Masayuki Yamato, Teruo Okano

**Affiliations:** a Institute of Advanced Biomedical Engineering and Science, Tokyo Women’s Medical University, Shinjuku, Japan; b Department of Materials Science and Technology, Graduate School of Advanced Engineering, Tokyo University of Science, Katsushika, Japan

**Keywords:** word, poly(*N*-isopropylacrylamide), thermoresponsive surface, polymer brush, terminal cationization, electrostatic interaction, cell adhesion, cell sheet, 20 Organic and soft materials (colloids, liquid crystals, gel, polymers), smart material, 211 scaffold / tissue engineering/drug delivery

## Abstract

A variety of poly(*N*-isopropylacrylamide) (PIPAAm)-grafted surfaces have been reported for temperature-controlled cell adhesion/detachment. However, the surfaces reported to date need further improvement to achieve good outcomes for both cell adhesion and detachment, which are inherently contradictory behaviors. This study investigated the effects of terminal cationization and length of grafted PIPAAm chains on temperature-dependent cell behavior. PIPAAm brushes with three chain lengths were constructed on glass coverslips via surface-initiated reversible addition-fragmentation chain transfer (RAFT) polymerization. Terminal substitution of the grafted PIPAAm chains with either monocationic trimethylammonium or nonionic isopropyl moieties was performed through the reduction of terminal RAFT-related groups and subsequent thiol-ene reaction with the corresponding acrylamide derivatives. Although the thermoresponsive properties of the PIPAAm brush surfaces were scarcely affected by the terminal functional moiety, the zeta potentials of the cationized PIPAAm surfaces were higher than those of the nonionized ones, both below and above the phase transition temperature of PIPAAm (30°C). When bovine endothelial cells were cultured on each surface at 37°C, the number of adherent cells decreased with longer PIPAAm. Notably, cell adhesion on the cationized PIPAAm surfaces was higher than that on the nonionized surfaces. This terminal effect on cell adhesion gradually weakened with increasing PIPAAm length. In particular, long-chain PIPAAm brushes virtually showed cell repellency even at 37°C, regardless of the termini. Interestingly, moderately long-chain PIPAAm brushes promoted cell detachment at 20°C, with negligible terminal electrostatic interruption. Consequently, both cell adhesion and detachment were successfully improved by choosing an appropriate PIPAAm length with terminal cationization.

## Graphical abstract

**Figure:**
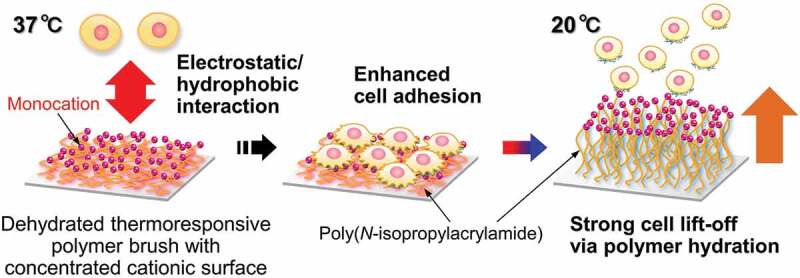

## 1. Introduction

Cell-based regenerative therapies for reproducing lost functions of human tissues and organs have attracted significant attention in recent decades [1–4]. We have previously proposed a promising method for human tissue reconstruction using cell-dense tissue monolayers, called ‘cell sheets’ [5,6]. Transplantation of cell sheets with depositing a biologically intact extracellular matrix (ECM) [7,8] enables for effective cell engraftment and treatment of the target damaged tissues without recourse to artificial materials, such as biodegradable scaffolds [9,10], or other operations, including suturing [11]. The fabrication of the cell sheets conventionally involves the seeding and confluent culture of suitable cells on thermoresponsive poly(*N*-isopropylacrylamide) (PIPAAm)-grafted surfaces at 37°C [5], following which, the cell sheet can be harvested by reducing the temperature below the PIPAAm’s lower critical solution temperature (LCST, approximately 30°C) for the rehydration and globule-to-coil change of thermoresponsive polymers [12].

First-generation thermoresponsive culture surfaces possess nanoscale cross-linked PIPAAm structures constructed by electron beam (EB) graft polymerization [13]. However, the EB method is not well-suited for precise control of the thickness, chain configuration, and density of the grafted polymers, which are key factors for temperature-controlled cell adhesion/detachment [14]. Many reports have described the preparation of well-defined PIPAAm surfaces via surface-initiated controlled radical polymerization [15,16] and physical polymer coatings [17,18]. Among these surfaces, densely grafted linear PIPAAm structures (PIPAAm brushes) readily tune thermoresponsive cell-surface interactions by varying the chain length and density of the grafted polymers [19]. Furthermore, an increase in the PIPAAm graft density generally reduces surface hydrophobicity and results in reduced cell-surface interaction, and thus cell detachment can be accelerated via low-temperature incubation [20]. However, a serious problem posed by this approach is that increased PIPAAm graft density often induces poor cell adhesion and proliferation.

To overcome this dilemma, thermoresponsive copolymer brushes, including cationic comonomers, have been designed to improve cell adhesion [21]. The introduction of cationic units to material surfaces promotes the absorption of cell adhesive molecules (e.g. fibronectin), and in turn, cell adhesion [22,23]. However, hydrophilic cationic comonomers often cause increases in the LCST of the original PIPAAm and change the hydrophobicity and extension/aggregation states of the thermoresponsive polymers [24]. Therefore, additional hydrophobic monomers must be introduced to tune the LCST and aggregation strength of the thermoresponsive cationic copolymers [21]. Moreover, random copolymerization does not achieve reproducible site-selective introduction of functional moieties. Therefore, there is a need for developing a method for the simple and effective functionalization of PIPAAm brushes.

Terminal functionalization has been used to introduce additional functional moieties to target polymers without changing their original characteristics [25,26]. This strategy can also be applied to linear polymer-grafted structures. We therefore reasoned that the outermost concentrated monocations on the dehydrated PIPAAm brushes would effectively stimulate cell adhesion without noticeable alterations in the thermoresponsive properties of PIPAAm. Based on this hypothesis, we constructed terminally monocationized PIPAAm brushes with various chain lengths on glass coverslips. We then investigated the effects of terminal monocation and length of the PIPAAm chains on temperature-dependent cellular behaviors.

## 2. Experimental section

### 2.1. Materials

*N*-Isopropylacrylamide (IPAAm) and (3-acrylamidopropyl)trimethylammonium chloride (APTAC) were provided by KJ Chemicals (Tokyo, Japan). IPAAm was recrystallized from hexane, and APTAC was used as received. 3-Aminopropyltriethoxysilane (APTES) was purchased from Shin-Etsu Chemical (Tokyo, Japan). 4-Cyano-4-[(dodecylsulfanylthiocarbonyl)sulfanyl]pentanoic acid (CDPA) as well as Dulbecco’s phosphate buffered saline without calcium chloride and magnesium chloride (DPBS) were obtained from Sigma-Aldrich (St. Louis, Missouri, USA). *N*-Hydroxysuccinimide (NHS), *N,N*’-dicyclohexylcarbodiimide (DCC), 4,4ʹ-azobis(4-cyanovaleric acid) (V-501), tris(2-carboxyethyl)phosphine hydrochloride (TCEP), diethyl ether, dehydrated toluene, methanol, acetone, dehydrated dichloromethane (DCM), and 1,4-dioxane were obtained from FUJIFILM Wako Pure Chemicals (Osaka, Japan) and used without further purification. Glass coverslips (24 mm × 50 mm, thickness: 0.2 mm) were purchased from Matsunami Glass (Osaka, Japan). Water used in this study was purified using a Milli-Q Synthesis A10 system (Millipore, Billerica, Massachusetts, USA).

### 2.2. Preparation of thermoresponsive PIPAAm brushes on glass coverslips

Amino-functionalized glass coverslips were prepared via a silane coupling reaction with APTES according to a previous report [27]. Chemical modification of dodecyltrithiocarbonate (DTTC) groups as chain transfer agents (CTAs) on glass coverslips was performed by a DCC coupling reaction between the surface-introduced amino groups and CDPA. Briefly, the amino-functionalized glass coverslips were immersed in DCM containing CDPA (5 mmol/L), DCC (7.5 mmol/L), and NHS (7.5 mmol/L) at room temperature (R.T.) for 24 h in the dark. Next, the CTA-immobilized coverslips (abbreviated as sCTA) were thoroughly washed with DCM and methanol, and then dried under vacuum.

**Scheme 1.**
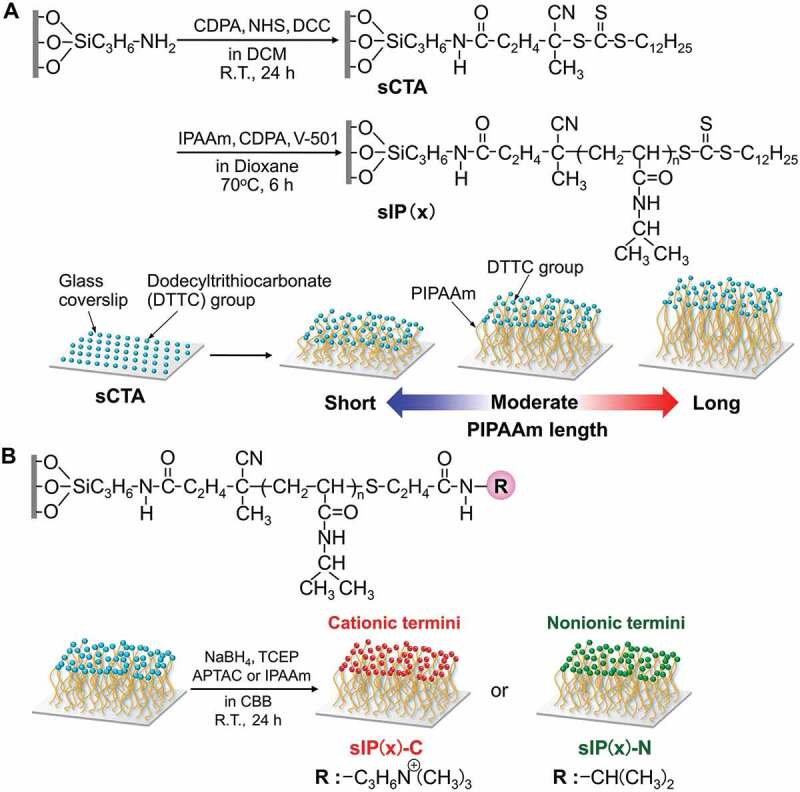
Synthetic pathway of (A) preparation of poly(*N*-isopropylacrylamide) (PIPAAm) brush surfaces and (B) the terminal conversion of PIPAAm brush with monocationic or nonionic acrylamide derivatives

PIPAAm brushes with various chain lengths on the glass coverslips were constructed via surface-initiated reversible addition-fragmentation chain transfer (SI-RAFT) polymerization using sCTA (Scheme 1(A)). The sCTA were immersed in 250 mL of 1,4-dioxane containing IPAAm (300, 375, or 450 mmol), CDPA (0.25 mmol), and V-501 (0.05 mmol), and then the reactive solution was deoxygenated by N_2_ gas bubbling for 1 h. The polymerization reaction was performed at 70°C for 6 h. The reaction was terminated by cooling in an ice bath. The reacted coverslips were immersed in acetone with magnetic stirring for 1 h followed by drying *in vacuo*. The obtained PIPAAm-grafted coverslips are abbreviated as sIP(*x*), where ‘*x*’ is the initial molar concentration of the IPAAm monomer in each polymerization.

In addition, the non-grafted free IPAAm polymer produced in each solution was collected by precipitation in an excess of diethyl ether to estimate the molecular weights of the grafted PIPAAm chains. The recovered free polymers were characterized by gel permeation chromatography (GPC). We used an HLC-8320GPC system (Tosoh, Tokyo, Japan) equipped with triple columns (TSKgel SuperAW2500, SuperAW3000, and SuperAW4000, Tosoh) and *N,N*-dimethylformamide containing 50 mmol/L LiCl as the eluent (flow rate: 0.6 mL/min, 40°C). The molecular weight and polydispersity index (PDI) of the PIPAAm molecules were calculated from a calibration curve based on poly(ethylene oxide) standards (Polysciences, Warrington, Pennsylvania, USA). The graft density of PIPAAm on the glass surfaces was estimated using the follow equation:

(1) $$Graft\;density\;=\;\frac{m_{p}N_{A}}{M_{n}}$$

where *m*_p_ is the weight of grafted PIPAAm per square centimeter, *N*_A_ is Avogadro’s number, and *M*_n_ is the number average molecular weight of the corresponding free PIPAAm determined by GPC.

### 2.3. Terminal conversion of thermoresponsive polymer brushes

RAFT-mediated PIPAAm brushes possess terminal CTA-derived thiocarbonylthio groups, which can be converted to thiol groups with reducing agents or primary amino compounds [28–30]. For the functionalization of PIPAAm termini via a thiol-ene reaction [28,30,31], the coverslips grafted with DTTC-terminated PIPAAm brushes were immersed in a carbonate/bicarbonate buffer (CBB) solution (0.1 mol/L, pH 9.8) including either monocationic APTAC or nonionic IPAAm (50 mmol/L), sodium borohydride (0.5 mol/L), and TCEP (15 mmol/L) for 24 h, as shown in Scheme 1(B). After the reaction, the glass coverslips were washed with water and acetone, and dried under vacuum. The PIPAAm brush surfaces with terminal cationic quaternary ammonium and nonionic isopropyl moieties are abbreviated as sIP(*x*)-C and sIP(*x*)-N, respectively.

### 2.4. Characterization of PIPAAm brush surfaces

The amounts of grafted PIPAAm on the glass surfaces were determined by attenuated total reflectance-Fourier transform infrared (ATR-FTIR) spectroscopy using a Nicolet 6700 spectrometer (Thermo Scientific, Waltham, Massachusetts, USA) equipped with a germanium ATR crystal (Harrick Scientific Corporation, Pleasantville, New York, USA). Briefly, the peak intensity derived from the amide carbonyl group of IPAAm (1650 cm^−1^) was normalized against the peak intensity of the Si-O bond of the glass substrate (1000 cm^−1^), and the grafted PIPAAm amounts were estimated from the intensity ratio using a calibration curve [27].

Temperature-dependent wettability changes of the terminally functionalized PIPAAm brush surfaces were determined using a drop shape analyzer DSA100 (KRÜSS, Hamburg, Germany) by the captive bubble method in DPBS. Prior to the measurements, the samples were immersed in DPBS at 20°C for 24 h. Air bubbles (5 μL) were placed onto the glass surfaces at specific temperatures ranging from 20°C to 37°C. The temperature of DPBS was controlled using a circulating thermostat (Lauda RE104) (Lauda, Lauda-Königshofen, Germany).

The surface zeta potentials of the terminally functionalized PIPAAm brush surfaces were determined using an electrophoretic light scattering device (ELS-8000) (Ohtsuka Electronics, Osaka, Japan). The surfaces were immersed in a 5 mmol/L KCl aqueous solution at 20°C for 24 h, and zeta potentials were measured at 20°C and 37°C using standard particles for the solid sample cell (Ohtsuka Electronics).

### 2.5. Cell experiments

Cell culture was conducted at 37°C or 20°C in a humidified atmosphere containing 5% CO_2_. Bovine carotid artery normal endothelial cells (Japanese Collection of Research Bioresources Cell Bank, Osaka, Japan) (a passage number: 20 − 25) were maintained in Dulbecco’s modified Eagle’s medium (Sigma-Aldrich) containing 10% fetal bovine serum (Japan Bioserum, Hiroshima, Japan) and 100 units/mL penicillin-100 μg/mL streptomycin (Sigma-Aldrich) on a 100-mm cell culture dish (Falcon 353003) (Corning, One Riverfront Plaza, New York, USA) at 37°C. The coverslip samples were cut in half (24 × 25 mm), and were independently placed on 35-mm dishes (not treated polystyrene, Falcon 351008) (Corning). To investigate cell adhesion and detachment profiles on the surfaces, 0.25% trypsin/EDTA-treated cells were seeded onto each dish at 1.5 × 10^4^ cells/cm^2^, cultured at 37°C for 24 h, and finally incubated at 20°C. The number of adherent cells was counted after specific time periods under a phase contrast microscope (ECLIPSE TE2000-U; Nikon, Tokyo, Japan). In the cell proliferation assay, seeded cells (1 × 10^4^ cells/cm^2^) were cultured at 37°C for 72 h. The cell doubling time (CDT) was calculated using the following equation:

(2) $$\left(CDT\right)=\left(t_{2}-t_{1}\right)log_{10}2/\left(log_{10}N_{2}-log_{10}N_{1}\right)$$

where *t_1_* = 24 h, *t_2_* = 72 h, and *N_1_* and *N_2_* are the number of adhering cells at 24 h and 72 h, respectively.

For harvesting cell sheets, cells were seeded onto the surfaces at a density of 1 × 10^5^ cells/cm^2^ and cultured at 37°C. After reaching cell confluency, the surfaces were incubated at 20°C, and the detachment behaviors of the cell sheets were observed visually.

### 2.6. Statistical analysis

Experimental data are expressed as the mean of at least three separate samples with standard deviation. Analyses of variance followed by two-tailed Student’s *t-test* were used to evaluate signiﬁcant differences among experimental groups. Statistical significance was set at *p* <_ _0.05.

## 3. Results and discussion

### 3.1. Preparation and characterization of terminally functionalized PIPAAm brushes

PIPAAm brushes are promising smart surfaces for effective control of adhesion/detachment of various types of cells, owing to the facile adjustment of chain length and density of the grafted polymer in response to a wide variety of cell adhesive properties. This study focused on the effects of terminal positive charge and chain length of PIPAAm brushes on the thermoresponsive cellular behaviors for creating advanced thermoresponsive polymer brushes. To prepare PIPAAm brushes with three controlled chain lengths, SI-RAFT polymerization was performed by changing the initial monomer concentrations using CTA-immobilized glass coverslips (Scheme 1(A)). RAFT polymerization allows for the synthesis of vinyl polymers with controlled molecular weights, and is applicable to a wide range of monomers under various experimental conditions [32,33]. Our previous studies demonstrated that RAFT-mediated polymer grafting to solid surfaces can be applied to the fabrication of thermoresponsive PIPAAm brushes with various controlled chain lengths [19]. Owing to the difficulty involved in determining the molecular weight of the grafted polymer chains, the indirect approach of characterizing the free polymers in the polymerization solutions has been employed instead [34]. Following this approach, unbound PIPAAm was obtained and evaluated by GPC. The number average molecular weight (*M*_n_) increased with increasing initial IPAAm concentrations (*M*_n_ of sIP(1.2): 43,000; sIP(1.5): 48,000; and sIP(1.8): 59,000) with relatively controlled polydispersity indexes of 1.5 − 1.6. Further, the amounts of grafted polymers increased with higher monomer concentration and ranged from 1.6 to 3.3 μg/cm^2^, as estimated by ATR-FTIR spectroscopy (Table 1). Using the *M*_n_ values of the corresponding polymers, the estimated graft density of PIPAAm on each surface was 0.2 − 0.3 chains/nm^2^. In previous studies, a polymer brush has been defined as closely packed polymer chains having a density of higher than 0.1 chains/nm^2^, with extending polymer conformation [35–37]. Therefore, these results demonstrate that closely packed PIPAAm brushes were successfully created on the glass coverslips.

**Table 1.** Characterization of various PIPAAm brush surfaces

Code	Amount of grafted PIPAAm (μg/cm^2^) ^*a*^	*M*_n_ (PDI) of free PIPAAm ^*b*^	PIPAAm density (chains/nm^2^) ^*c*^
sIP(1.2)-C	1.6 ±_ _0.4	4.3 × 10^4^ (1.50)	0.23
sIP(1.2)-N	1.6 ±_ _0.2		0.23
sIP(1.5)-C	2.1 ±_ _0.1	4.8 × 10^4^ (1.60)	0.26
sIP(1.5)-N	2.2 ±_ _0.3		0.28
sIP(1.8)-C	3.2 ±_ _0.2	5.9 × 10^4^ (1.54)	0.32
sIP(1.8)-N	3.3 ±_ _0.2		0.33

^a^Estimated by ATR-FTIR

^b^Measured by GPC.

^c^Calculated using Equation (1).

Prior to starting this study, we investigated the effect of terminal monocation on the thermoresponsive properties of PIPAAm. The similarity of the chemical structures of the terminal groups and the PIPAAm backbone as well as the presence of a small number of terminal molar components led to difficulties in estimating the terminal substitution degrees of the polymer chains. As a result, we investigated the differences between the terminal absorbances and molecular weight distributions of the PIPAAm molecules before and after terminal substitution. The absorbance derived from the terminal DTTC moieties disappeared after terminal substitution (Figure S1 in the Supplemental material). In addition, the molecular weight distributions of PIPAAm molecules changed negligibly before and after terminal substitution (Figure S2 in the Supplemental material). These results demonstrate that the terminal reduction and substitution of the PIPAAm chains proceeded effectively with minimal side reactions. As described in Figure S3 and Table S1 of the Supplemental material, the LCST of trimethylammonium-terminated PIPAAm was comparable to that of isopropyl-terminated PIPAAm, with negligible alteration of aggregation behavior regardless of polymer molecular weights (43,000–59,000).

**Figure 1.**
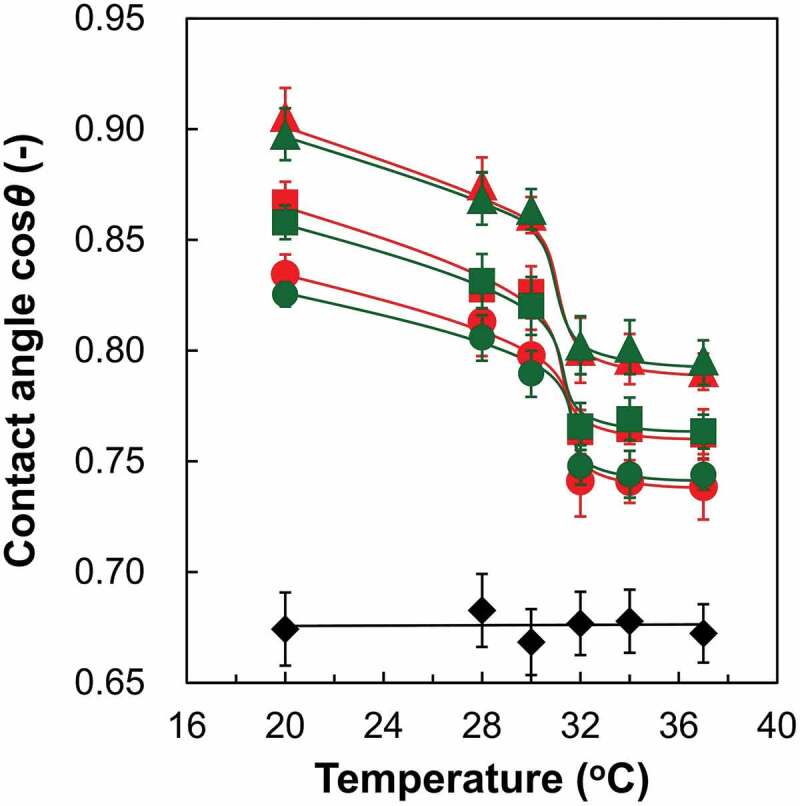
Temperature-dependent static water contact angle changes of terminally functionalized PIPAAm brush surfaces with various polymer chain lengths. CTA-immobilized surface (sCTA): closed diamond; sIP(1.2)-C: red circle; sIP(1.2)-N: green circle; sIP(1.5)-C: red square; sIP(1.5)-N: green square; sIP(1.8)-C: red triangle; sIP(1.8)-N: green triangle

According to previous studies, surface water wettability plays an important role in the thermoresponsive regulation of cell adhesion to, and detachment from, PIPAAm-grafted culture surfaces [20]. Notably, the chain length of grafted PIPAAm is a critical factor in determining the surface wettability [15,19]. Therefore, temperature-dependent surface wettability changes of various PIPAAm brush surfaces were investigated by static water contact angle measurements at temperatures ranging from 20°C to 37°C. Although the DTTC-immobilized surfaces (sCTA) were moderately hydrophobic, PIPAAm grafting resulted in significant increases in surface hydrophilicity, as shown in Figure 1. Further, surface hydrophobicity increased with the length of the PIPAAm chains, which is probably owing to the increase in the surface component of the acrylamide derivatives. Significantly, the PIPAAm brush surfaces exhibited temperature-dependent changes in surface wettability regardless of polymer chain length and terminal functional moiety. The values of cosθ of the PIPAAm surfaces decreased gradually upon heating from 20°C, and significantly changed near the LCST of PIPAAm (29 − 31°C). Notably, the difference in terminal functionality between cationic and nonionic groups did not significantly affect the surface wettability and thermoresponsive phase transition behavior of the PIPAAm brush surfaces. Our previous studies described that the terminal hydrophobic group of the PIPAAm brush clearly shifted the thermoresponsive phase transition to a lower temperature due to the promotion of polymer dehydration [27,38]. However, the influence of the hydrophilic electrostatic termini on the phase transition of the grafted PIPAAm chains was negligible. Overall, the thermoresponsive behaviors and water-wettability of the PIPAAm brush surfaces were dominated by the original characteristics of the grafted polymers, and independent of the terminal functional groups.

**Figure 2.**
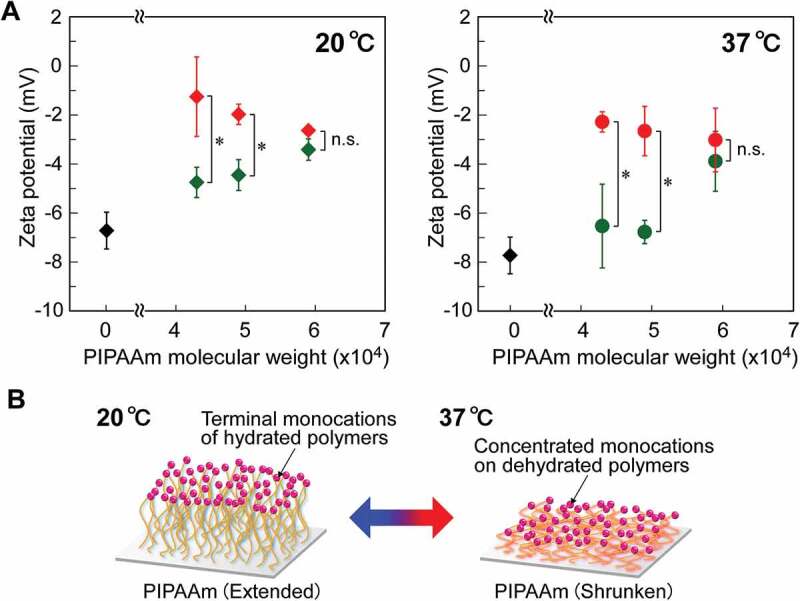
(A) Zeta potentials of terminally functionalized PIPAAm brush surfaces with various PIPAAm molecular weights at 20°C and 37°C. Red and green marks represent the terminally cationized and nonionized PIPAAm brushes, respectively, while black markers represent CTA-immobilized surfaces (sCTAs). n.s.: not significant and *p < 0.05. (B) Schematic illustration of temperature-dependent structural changes of the terminally cationized PIPAAm brush surface

The surface zeta potentials of the PIPAAm brushes on the glass coverslips were investigated at temperatures below and above the LCST of PIPAAm (20°C and 37°C, respectively) via the electro-osmosis method, as shown in Figure 2(A). Non-polymer-grafted sCTA exhibited temperature-independent and negative potentials (values of −6.7 mV and −7.7 mV at 20°C and 37°C, respectively). Considering that the zeta potential of the bare glass surface is approximately −25 mV [39], the potential of the sCTA surfaces was probably determined by the balance between the number of exposed silanol groups (negatively charged) and unreacted amino groups (positively charged) on the glass surfaces in contact with 5 mmol/L KCl aqueous solution. These negative potentials were significantly decreased by grafting PIPAAm onto the coverslip. In particular, the decrease in negative potential became larger with longer PIPAAm chains. This result indicates that the closely packed PIPAAm segments effectively covered glass surfaces and shielded the charged groups. Importantly, for the same PIPAAm length, the zeta potentials of the cationic-terminated PIPAAm brush surfaces were much higher than those of the nonionic ones at 20°C (for example, cationic-terminated sIP(1.2)-C: −1.3 ± 1.6 mV; nonionic-terminated sIP(1.2)-N: −4.8 ± 0.6 mV, *p* < 0.05). These results clearly demonstrate the successful introduction of terminal monocations into the grafted PIPAAm chains. The influence of cationic termini diminished with increasing PIPAAm chain length (cationic-terminated sIP(1.8)-C: −2.6 ± 0.1 mV; nonionic-terminated sIP(1.8)-N: −3.4 ± 0.4 mV, *p* > 0.05). Previous studies reported that closely packed and hydrated polymer brushes show the expanding conformation of polymers due to the volume exclusion effect [35,36]. In this study, the PIPAAm brushes were considered to possess relatively controlled polymer lengths based on the GPC results of the recovered free polymers. Owing to the unique grafting structures with the packing of comparable polymers, the terminal monocations were probably concentrated at the periphery of the PIPAAm brushes, causing significant potential changes. The cationic concentrations on the polymer brushes might be disturbed by the increase in the molecular length of PIPAAm, owing to larger chain mobility.

At the temperature above the LCST (37°C), the cationized PIPAAm brushes exhibited significantly higher zeta potentials than the nonionized surfaces (for example, cationic-terminated sIP(1.2)-C: −2.3 ± 0.4 mV; nonionic-terminated sIP(1.2)-N: −6.5 ± 1.7 mV, *p* < 0.05), even when the polymers were dehydrated and shrunken. This is likely related to the PIPAAm brush structures. The densely packed polymer structure may have prevented terminal monocations from being obscured within the polymer layers and maintained the positive charges at the surface (Figure 2(B)). The concentration of the monocations at the surfaces of terminally functionalized PIPAAm brushes is also desirable for effective interactions with proteins and cells.

The potentials of all PIPAAm brush surfaces at 37°C were more negative than those at 20°C. The shifts to negative values were probably caused by structural changes in the grafted PIPAAm. The zeta potential has been defined as the electrical potential at the slipping plane, where is a hydrodynamic shear surface separating the mobile fluid away from the interface. When the temperature rose above the LCST, the plane of each surface approached the glass interface via the coil-to-globule transition of the grafted PIPAAm, resulting in a larger influence of negative charges derived from the silanol groups. In addition, the magnitude of the decreases in potential became smaller with longer PIPAAm chains. This temperature-dependent shift of zeta potential is similar to a previous report describing PIPAAm-corona nanoparticles [40]. These results indicate that longer PIPAAm chains effectively masked the negatively charged glass surface even in the shrunken compact conformation.

### 3.2. Cell adhesion and detachment assay

**Figure 3.**
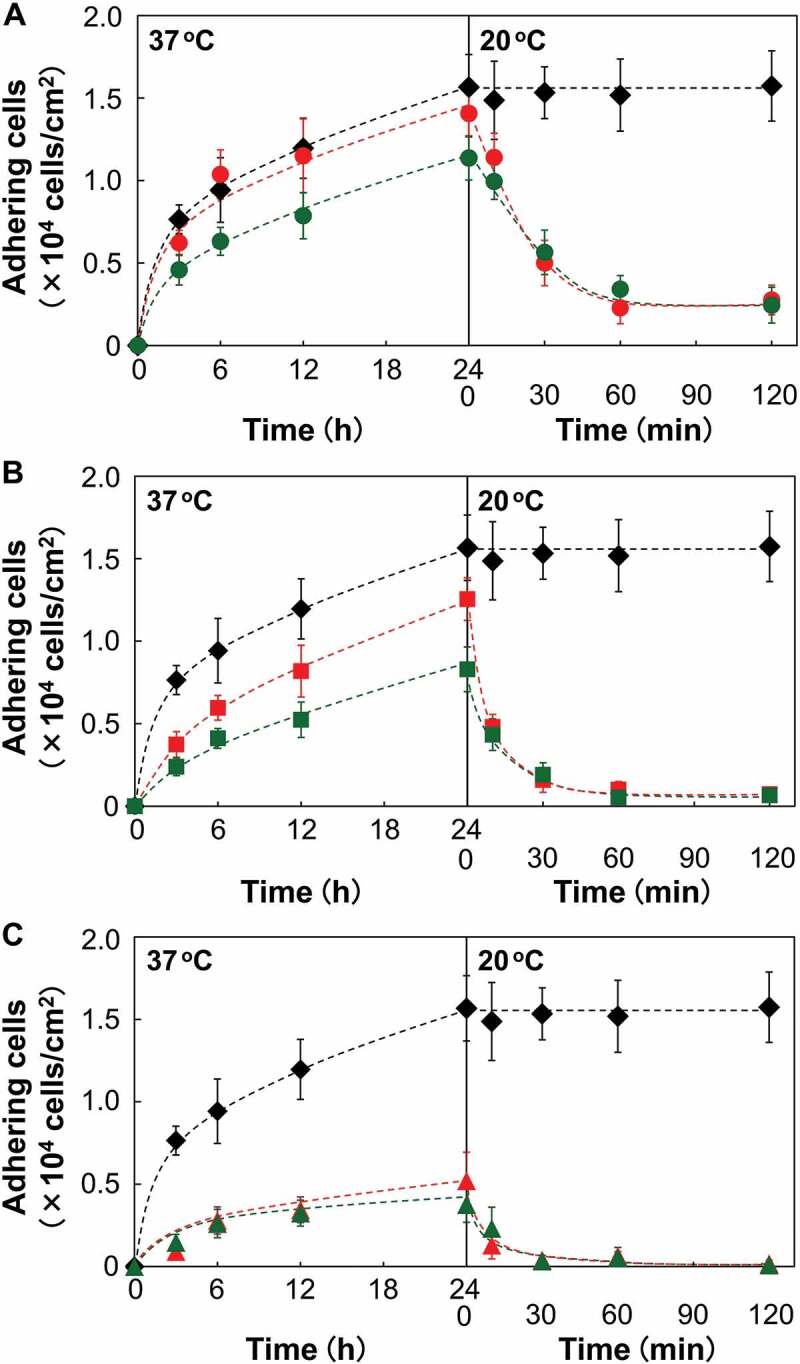
Cell adhesion and detachment profiles on the cationic- or nonionic-terminated PIPAAm brush surfaces. Cells (seeding density: 1.5 × 10^4^ cells/cm^2^) were incubated at 37°C for 24 h, and then, incubated again at 20°C for 120 min. (A) sIP(1.2) surfaces, (B) sIP(1.5) surfaces, and (C) sIP(1.8) surfaces. Red and green markers represent the terminally cationized and nonionized PIPAAm brushes, respectively, while black markers represent CTA-immobilized surfaces (sCTAs)

Cell culture experiments on various PIPAAm brush surfaces were performed to investigate the influence of terminal functional moiety and chain length of grafted polymers on cellular behavior at temperatures below and above the LCST of PIPAAm (37°C and 20°C, respectively). At 37°C, the efficiency of cell adhesion was significantly affected by the physicochemical properties of the grafted PIPAAm chains. The molecular length and mobility of linear PIPAAm chains on solid surfaces are known to be key factors in the thermal control of interactions with cells and proteins [18]. The sCTA demonstrated a strong cell adhesive property owing to its surface hydrophobicity, while cell adhesion on the PIPAAm brush surfaces was affected by the PIPAAm length. The number of adherent cells decreased with increasing PIPAAm chain length (Figure 3, left). As mentioned in the discussion about the contact angles of the PIPAAm brush surfaces, longer PIPAAm chains promote surface hydrophilicity. It follows that the reduced cell adhesion is related to the surface hydrophilization as a result of PIPAAm grafting. A number of studies have addressed the relationship between cell behavior and culture substrates with various surface factors including hydrophobicity, roughness, and electrostatic charge [41,42]. An increase in surface hydrophilicity leads to a decrease in the adsorption of cell adhesion proteins, which mediate cell adhesion to material surfaces. In addition to the surface hydrophilicity, previous studies have also reported that long PIPAAm chains possess large molecular motion and loose aggregation in their dehydrated state, and reduce cell-surface interactions [18,19]. In fact, the sIP(1.8) possessing long-chain PIPAAm demonstrated small number of adherent cells even at 37°C, above the LCST.

For the short- and moderately long-chain PIPAAm brush surfaces (sIP(1.2) and sIP(1.5)), the cell adhesion efficiency was significantly improved by terminal cationization (Figure 3, left). For example, the sIP(1.2)-C and sIP(1.5)-C surfaces showed enhancements in cell adhesion of approximately 23% and 51%, respectively, over the corresponding nonionized surfaces, sIP(1.2)-N and sIP(1.5)-N. Interestingly, the adhesive profile of the cationized surfaces with moderate PIPAAm length (sIP(1.5)-C) was comparable to that of the nonionized short-chain PIPAAm surfaces (sIP(1.2)-N). Previous studies have reported that electrostatic functional groups concentrated on self-assembled monolayers promote protein adsorption and cell adhesion [43]. According to the results of the zeta potential study, the improvement in cell adhesion could be attributed to the concentrated monocations at the periphery of the dehydrated PIPAAm brushes. Therefore, it is likely that the promoted cell adhesion was caused by the enhanced adsorption of adhesive proteins, including fibronectin (p*I*: 5.5–6.0) [22]. However, the lack of cell adhesion on the sIP(1.8)-N surface was not noticeably improved by terminal cationization. These results strongly indicate that optimization of the PIPAAm chain length is a key factor in maximizing the terminal cationic effect on cell adhesion.

**Figure 4.**
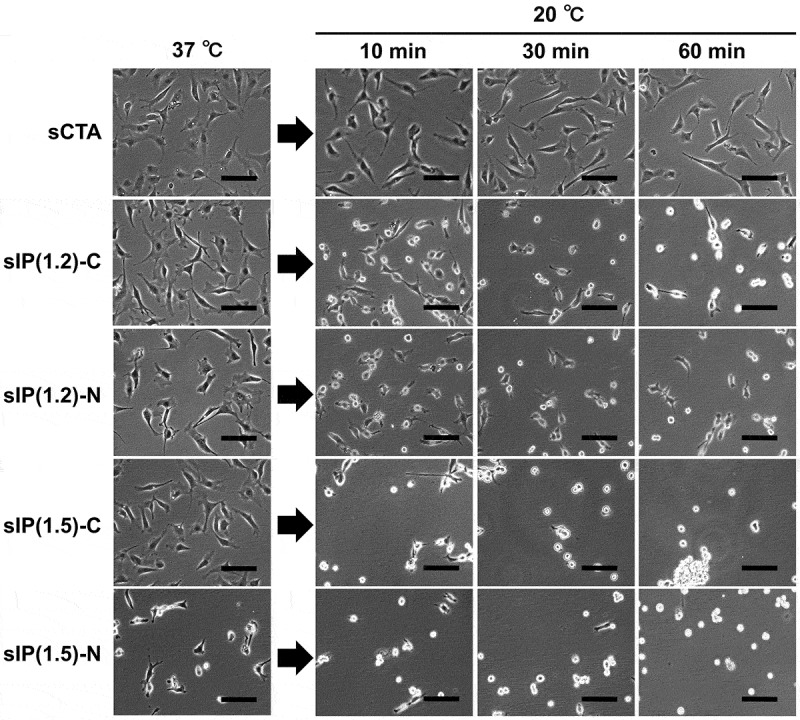
Optical microphotographs of adhering cells at 37°C for 24 h, and detached cells from the surfaces at 20°C. Scale bar: 100 μm

**Figure 5.**
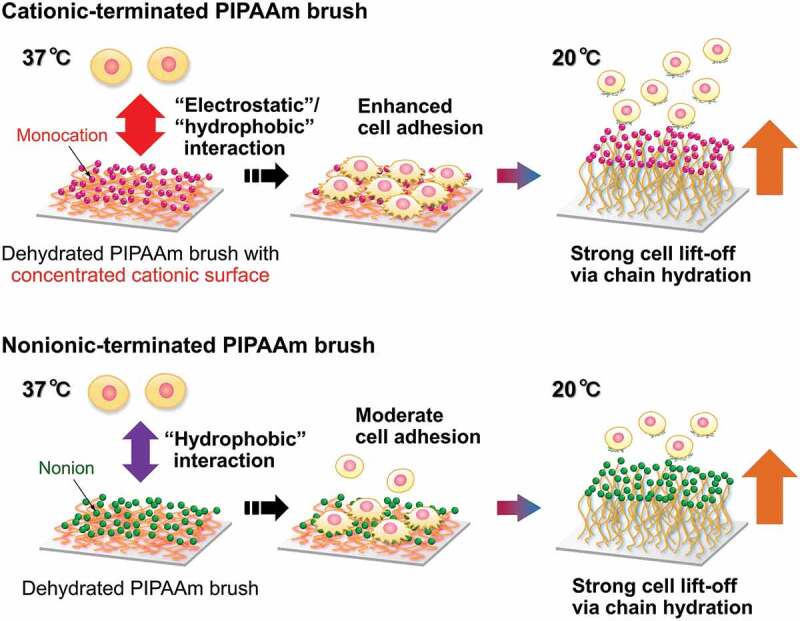
Schematic illustration of cell adhesion and detachment on the terminally functionalized PIPAAm brush surfaces

Cultured cells were exposed to a temperature of 20°C to assess temperature-dependent cell detachment profiles from various PIPAAm brush surfaces. Cell detachment via low-temperature treatment also depends on the PIPAAm chain length. Non-polymer-grafted sCTA surfaces did not release cells, while all PIPAAm brush surfaces demonstrated spontaneous cell detachment (Figures 3 and 4). In particular, the moderate and long PIPAAm-grafted surfaces effectively lifted off the adherent cells and more than 90% of the cells detached from the surfaces within 30 min (Figure 3(B,C)). The sIP(1.2), with the short PIPAAm chains, terminated cell detachment while maintaining approximately 20% adherent cells even after incubation for 120 min (Figure 3(A)). In addition to the influence of surface hydrophilization, longer PIPAAm chains effectively accelerated cell detachment because a larger scale of the globule-to-coil transition induced a dynamic cell-surface affinity change [15,18,19]. Even though the terminal cations significantly promoted cell adhesion via electrostatic effects, delayed cell detachment from the cationized surfaces was not observed regardless of PIPAAm length (Figure 3, right). This negligible terminal effect on cell detachment was possibly due to the rehydration and conformational changes of the PIPAAm chains, which readily release the cells from the surfaces and overcome the outermost electrostatic interactions. Consequently, the outermost monocations of the dehydrated PIPAAm brushes effectively promoted cell adhesion via electrostatic interactions, but did not noticeably interrupt cell release from the surfaces (Figure 5). This unique property of terminal cationic functionality with mild electrostatic cellular interactions would be useful for a rapid cell capture and release system in cell separation as well as thermoresponsive cell cultures. In this decade, thermoresponsive cationic brush surfaces have been investigated to develop smart cell separation systems for the purification of target cells (e.g. mesenchymal stem cells) from cell mixture without cell labelling [44,45]. These cell separations are known to be achieved because of the differences between individual cellular properties, including zeta potentials and cell adhesion/detachment behaviors.

### 3.3. Cell proliferation assay

**Figure 6.**
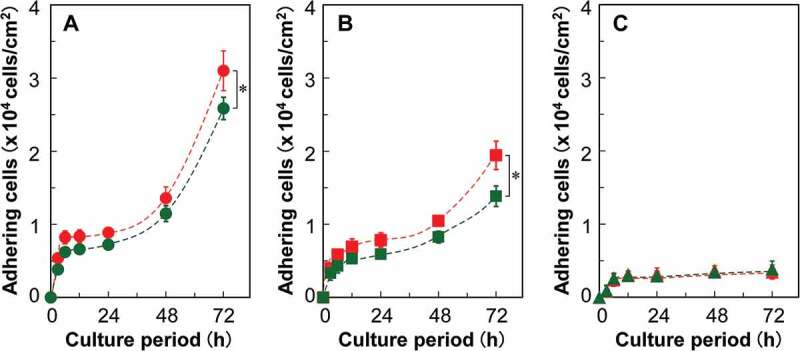
Cell proliferation on the cationic- or nonionic-terminated PIPAAm brush surfaces. Cells (seeding density: 1 × 10^4^ cells/cm^2^) were incubated at 37°C. (A) sIP(1.2) surfaces, (B) sIP(1.5) surfaces, and (C) sIP(1.8) surfaces. Red and green markers represent the terminally cationized and nonionized PIPAAm brushes, respectively. *p < 0.05

Cell proliferation profiles on various PIPAAm brush surfaces were investigated to determine the effects of the terminal functional moiety and polymer length (Figure 6). An increase in PIPAAm length diminished the efficiency of cell proliferation. The reduction in proliferation is believed to be related to the surface hydrophilization of the PIPAAm brushes. As previously mentioned in the discussion for the static contact angle study, longer PIPAAm chains promoted a more hydrophilic surface because of the increase in the number of polyacrylamide derivatives. In addition, on the sIP(1.2)-C and sIP(1.5)-C surfaces as the cationized PIPAAm brushes, the number of proliferated cells was larger than those on the nonionized surfaces. The CDT for the short-chain PIPAAm brushes (sIP(1.2)) was found to be approximately 23 h, regardless of the terminal functional moiety. This is due to the good cell adhesive properties in both cases. However, terminal cationization reduced the CDT for sIP(1.5) by 2 h (CDT of sIP(1.5)-C: 24.3 h; sIP(1.5)-N: 26.2 h). The reduction of CDT was attributed to the effective and stable cell interactions with the positively charged polymer surfaces. Overall, cell proliferation was dominated by the graft density of PIPAAm. The terminal cationization of PIPAAm brushes promoted the initial cell adhesion, but may not have significantly altered the biological functions of the adhering cells. In general, large amounts of cationic moieties on material surfaces often trigger toxic actions to cells via electrostatically induced disruption of cellular membranes [46]. However, the evenly distributed and concentrated monocations on the dehydrated PIPAAm brushes did not show any noticeable cytotoxicity, while maintaining consistent cell proliferation due to the small amounts of cationic units. On the other hand, the introduction of cationic termini made no observable difference to proliferation of the cells adhering on the sIP(1.8) surfaces grafted with long-chain PIPAAm. This loss of terminally cationic effects strongly agreed with the results of the surface zeta potential study, i.e. long PIPAAm chains might reduce the focal adhesions and result in a lack of proliferation owing to the advanced surface hydrophilization and less cationic effect.

### 3.4. Fabrication of cell sheets

**Figure 7.**
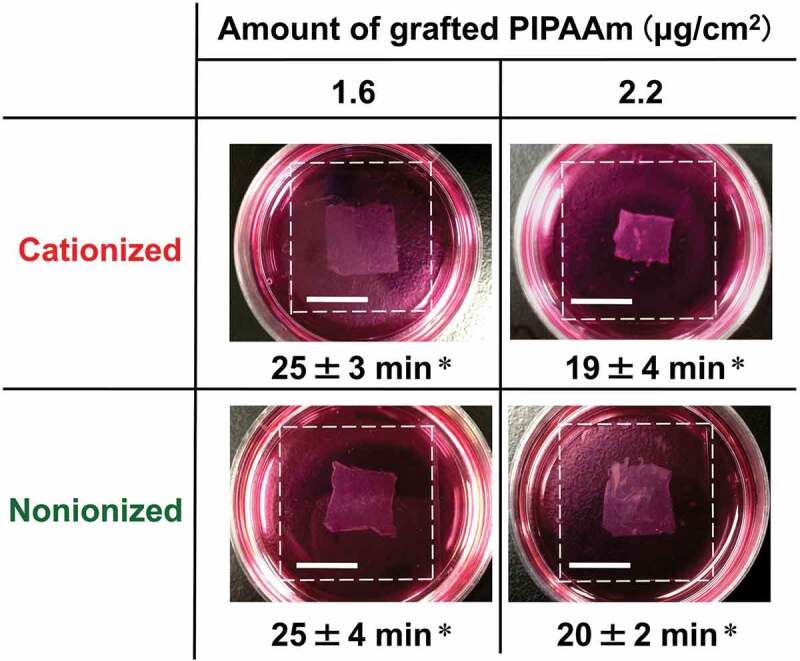
Photographs of cell sheets from the terminally functionalized PIPAAm brushes. The dashed white lines show the edges of the glass surfaces grafted with PIPAAm brushes. Scale bar: 1 cm. *Average of the time for completed detachment of three samples with standard deviation

To produce cell sheets by temperature changes, cultured cells are required to reach confluency on thermoresponsive PIPAAm surfaces. For the surfaces grafted with the short- and moderately long-chain PIPAAm molecules (sIP(1.2) and sIP(1.5)), confluent cell cultures were completed within 4 days, regardless of the terminal functional moieties. In contrast, the adherent cells on neither of the functionalized sIP(1.8) with long-chain PIPAAm reached confluency, owing to considerably low cell adhesion and growth properties even after 1 week (Figure S4 in the Supplemental material). After attainment of cell confluency on the sIP(1.2) and sIP(1.5) surfaces with the cationized and nonionized polymer termini, the surfaces were incubated at 20°C, for visual observation of the detachment behavior of the cell sheets. As shown in Figure 7, low-temperature treatment allowed for the harvesting of square-shaped cell sheets from all surfaces. The detached cell sheets possessed biologically intact cell-cell junctions, and their sizes were smaller than those of the thermoresponsive glass coverslips owing to the cytoskeletal rearrangement of individual cells in the cell sheet. Our previous study demonstrated that detached cell sheets from PIPAAm brush surfaces maintained ECM proteins, including fibronectin on their basal side [19]. On the other hand, the chain lengths of the grafted PIPAAm significantly affected the detachment profiles of the cell sheets. The time period of spontaneous sheet detachment became shorter with longer PIPAAm chains: the average times were 25 min for sIP(1.2) and 19–20 min for sIP(1.5). Owing to the impact of the terminal functional effect on cell detachment described earlier, cell sheets could be harvested from the PIPAAm brush surfaces with negligible interruption by the terminal electrostatic moieties. Overall, good outcomes for both cell adhesion and detachment in cell sheet fabrication were successfully achieved by choosing an appropriate PIPAAm length with simple terminal cationization. This surface design of terminally cationized PIPAAm brush surfaces could be used to prepare cell sheets from various low-surface-adhesive cells (e.g. human umbilical vein endothelial cells and hepatocytes) through the effective support of cell adhesion and rapid detachment by temperature changes.

## 4. Conclusions

This study reported the surface design of terminally monocationized PIPAAm brush surfaces to achieve robust outcomes for both adhesion and detachment of cells by changing the temperature. The influence of the terminal electrostatic functional moiety on the thermoresponsive surface properties of the PIPAAm brushes was negligible, while the chain length and terminal monocation of PIPAAm brushes considerably affected the thermoresponsive cellular behaviors. Longer PIPAAm chains reduced cell adhesion because of the increased surface hydrophilicity but accelerated cell detachment from the thermoresponsive surfaces. Interestingly, cationized PIPAAm surfaces showed markedly high cell adhesion compared with nonionized ones. This is likely owing to the monocations concentrated at the periphery of the dehydrated PIPAAm brushes. Importantly, terminal electrostatic functional moieties did not noticeably affect the lifting off of the adherent cells because cell detachment was mainly caused by the rehydration and globule-to-coil change of the grafted polymers. Consequently, by choosing an appropriate PIPAAm length with terminal cationization, the functionalized PIPAAm brush surfaces achieved both improvement of cell culture as well as rapid cell sheet detachment. Our findings pertaining to the simple terminal functionalization of thermoresponsive polymer brushes may contribute to various biomedical applications in smart cell culture and cell separation.
